# A prediction model for recurrence after translabyrinthine surgery for vestibular schwannoma: toward personalized postoperative surveillance

**DOI:** 10.1007/s00405-021-07244-z

**Published:** 2022-01-12

**Authors:** Nick P. de Boer, Stefan Böhringer, Radboud W. Koot, Martijn J. A. Malessy, Andel G. L. van der Mey, Jeroen C. Jansen, Erik F. Hensen

**Affiliations:** 1 Department of Otorhinolaryngology/Head and Neck Surgery, Leiden University Medical Center, Albinusdreef 2, 2333 ZA Leiden, The Netherlands; 2 Department of Biomedical Data Sciences, Leiden University Medical Center, Leiden, The Netherlands; 3 Department of Neurosurgery, Leiden University Medical Center, Leiden, The Netherlands

**Keywords:** Vestibular schwannoma, Prediction model, Recurrence, Translabyrinthine surgery

## Abstract

**Purpose:**

The aim of this study is to compute and validate a statistical predictive model for the risk of recurrence, defined as regrowth of tumor necessitating salvage treatment, after translabyrinthine removal of vestibular schwannomas to individualize postoperative surveillance.

**Methods:**

The multivariable predictive model for risk of recurrence was based on retrospectively collected patient data between 1995 and 2017 at a tertiary referral center. To assess for internal validity of the prediction model tenfold cross-validation was performed. A ‘low’ calculated risk of recurrence in this study was set at < 1%, based on clinical criteria and expert opinion.

**Results:**

A total of 596 patients with 33 recurrences (5.5%) were included for analysis. The final prediction model consisted of the predictors ‘age at time of surgery’, ‘preoperative tumor growth’ and ‘first postoperative MRI outcome’. The area under the receiver operating curve of the prediction model was 89%, with a C-index of 0.686 (95% CI 0.614–0.796) after cross-validation. The predicted probability for risk of recurrence was low (< 1%) in 373 patients (63%). The earliest recurrence in these low-risk patients was detected at 46 months after surgery.

**Conclusion:**

This study presents a well-performing prediction model for the risk of recurrence after translabyrinthine surgery for vestibular schwannoma. The prediction model can be used to tailor the postoperative surveillance to the estimated risk of recurrence of individual patients. It seems that especially in patients with an estimated low risk of recurrence, the interval between the first and second postoperative MRI can be safely prolonged.

## Introduction

The aim of treatment of patients with a vestibular schwannoma is to obtain long-term tumor control while keeping functions intact as much as possible. Depending on tumor characteristics such as size and progression, and factors such as functional hearing, patient’s age and preference, three management strategies can be opted for: active surveillance, radiotherapy and surgery. Different surgical approaches exist, such as the retrosigmoidal, translabyrinthine and subtemporal approach, of which the retrosigmoidal and translabyrinthine approaches are the most widely used techniques. When surgical resection is performed, a less than total tumor resection can be opted for to reduce the risk of postoperative facial nerve paresis. The inherent downside of a less than total tumor resection is the risk for regrowth of the tumor remnant, sometimes necessitating salvage treatment. Even after perceived total tumor resections, recurrence of tumors has been reported [1–5]. The extent of tumor resection as judged by the surgeon is theoretically a predictor for the risk of recurrence [3]. However, this predictor is difficult to use due to the varying ways in which the extent of tumor removal is reported [2–4, 6, 7]. In addition, discrepancies are found between the extent of resection estimated at the end of surgery and what is actually seen on the first postoperative magnetic resonance imaging (MRI) [6, 8]. Because of the risk of regrowth of residual tumor, postoperative surveillance with serial MRI is routinely performed. Currently, there is no consensus regarding the timing and frequency of MRI scanning following surgery [7, 9, 10]. Ideally, a follow-up MR imaging scheme should be tailored to the risk of individual patients for growth of residual tumor after surgery, especially regrowth necessitating salvage treatment.

The primary aim of this study is to compute and validate a statistical predictive model for the risk of recurrence, defined as regrowth of tumor or residual tumor growth necessitating salvage treatment, after translabyrinthine removal of vestibular schwannomas. With reliable prediction of the risk of recurrence and the time to recurrence should one occur, an individualized postoperative follow-up scheme becomes possible, on the one hand minimizing the number of MRI investigations needed while on the other allowing for timely reintervention when necessary.

## Materials and methods

### Patient population

A multivariable predictive model for risk of recurrence was fitted on retrospectively collected patient data between 1995 and 2017. The data from all sporadic, unilateral vestibular schwannomas operated via the translabyrinthine approach were obtained from the patients’ medical files and records of the Skull Base Center Leiden, a dedicated multidisciplinary team at the Leiden University Medical Center, Leiden, The Netherlands. This patient cohort has been described previously, focusing on prognostic factors for the outcome of surgery and facial nerve function, with consent of the local medical ethical committee [3]. The current study was reported following the statement for the Transparent Reporting of a multivariable prediction model for Individual Prognosis Or Diagnosis (TRIPOD) [11].

### Potential predictors

The potential predictive factors for tumor recurrence after surgery were selected based on a previous study of this patient cohort, the literature and expert opinion [3, 7, 12–14]. The factors investigated for inclusion in the model were: ‘age at time of surgery’, ‘gender’, ‘tumor size’, ‘cystic degeneration’, ‘preoperative tumor growth’, ‘extent of tumor resection’ and ‘first postoperative MRI outcome’. The tumor size was measured on the most recent preoperative MRI scan. These measurements were expressed linearly in millimeters, as the largest extrameatal diameter, excluding the intrameatal part. The presence of cystic degeneration was defined as the occurrence of inhomogeneous areas within the tumor on T2 and/or contrast-enhanced T1 MR imaging. Preoperative tumor progression was defined as an increase of more than 2 mm of the largest extrameatal diameter on sequential MR imaging within 12 months. Tumors that did not meet this criterion were categorized as stable (or regressive) [15]. The extent of tumor resection assessed by the surgeon at the end of surgery was defined as total (all tumor tissue removed), near total (less than 2% of original tumor remaining), or subtotal (removal of as much of the tumor as possible, with more than 2% of original tumor remaining). At the Skull Base Center Leiden, patients are routinely scanned with MRI at 6–12 months postoperatively to assess the presence of residual tumor with subsequent yearly MRI scanning to document potential residual tumor (re)growth, increasing the interval when multiple postoperative MR investigations show no or stable tumor residue. Gadolinium contrast enhancement patterns were categorized by experienced neuroradiologists as residual tumor, no residual tumor or uncertain enhancement. The prediction end point was the interval from the date of surgery to the date of the MRI on which the recurrence was detected or the date of the most recent MRI if growth was absent. A recurrence was defined as regrowth of tumor or progression of residual tumor on postoperative T2 weighted and/or gadolinium-enhanced T1-weighted MR imaging requiring salvage treatment, being either a second surgery or radiotherapy.

### Statistical analysis

Cox regression analyses were performed to evaluate potential predictive factors and to quantify their relative importance in predicting tumor recurrence, with recurrence-free survival as outcome and time interval defined as time since surgery in months. Missing data regarding the potential predictive factors were assumed to be missing completely at random, and imputation of missing data was not performed. Hazard ratios (HR) were used to express the strength of the association with 95% confidence intervals (CI). A value of *p* < 0.05 was considered statistically significant. Cohen’s kappa (*κ*) was used for inter-observation reliability between categorical variables [16]. A Cohen’s kappa value of 0 indicates agreement based on random association and a value of 1 indicates perfect agreement [17].

Selection of the factors for the development of the prediction model was based on the discriminating ability of different models as quantified by computing a receiver operating curve (ROC) and calculating the area under the curve (AUC). The AUC ranges between 50% (no added discriminating ability beyond a coinflip) and 100% (perfect discrimination). To prevent overfitting, the one-variable-per-10-events-rule, i.e., recurrences, was applied [18]. The final prediction model was assessed in terms of discriminating ability (C-index) and model validity (calibration, cross-validated C-index). The C-index measures whether a higher risk score, as computed by the model, corresponds to an earlier event probability when comparing two patients. The C-index ranges between 0 and 1, with a higher value corresponding to a better discriminative ability. Model calibration was performed to assess overfitting, whereby the model was fitted on part of the data and applied to unseen data (data-splitting) and a model only including the risk scores is evaluated in the unseen data. This step also assesses the internal validity of the model. Because of the low incidence of a recurrent vestibular schwannoma, tenfold cross-validation was used for model calibration, which optimally uses the full data as compared to performing a single data split [19].

As previously reported, the overall risk of recurrence in this cohort was 5.5% [3]. Based on clinical criteria and expert opinion, we defined a risk calculation of less than 1% as ‘low risk’ of recurrence.

Statistical data analyses were performed using IBM SPSS Statistics for Windows, version 25.0.3 (Armonk, NY: IBM Corp.), and R version 3.5.1 (The R Foundation for Statistical Computing, Vienna, Austria).

## Results

### Patient characteristics

A total of 661 patients with a unilateral vestibular schwannoma underwent surgery using the translabyrinthine approach between 1995 and 2017. A total of 65 patients were excluded, either because of incomplete radiological follow-up (*n* = 32), a combined or extended surgical approach (*n* = 14), previous radiotherapy (*n* = 12) or neurofibromatosis type 2 (*n* = 7). After applying these exclusion criteria, a cohort of 596 patients was included for the development of the predictive model. In total, 33 patients (5.5%) had a recurrent tumor. The clinical characteristics of the patients with and without a recurrence are shown in Table 1. At the time of salvage treatment, the average age of the patients suffering from a recurrence was 50 years (range 24–77 years) and the mean tumor size, measured at the MRI prior to the salvage treatment, of the recurrences in these patients was 19 mm (in maximal diameter). Missing data only occurred in the variable ‘cystic degeneration’ (*n* = 16, 3%).

**Table 1:** Patient characteristics

	All patients, *n* = 596	Recurrence free, *n* = 563	Recurrence, *n* = 33
Mean age at surgery, years (SD, range)	53 (12, 15–81)	53 (12, 15–81)	46 (12, 21–70)
Gender, *n* (%)			
Women	332 (56)	314 (56)	18 (55)
Men	264 (44)	249 (44)	15 (45)
Mean tumor size, mm (SD, range)	21 (10, 2–66)	21 (11, 2–66)	22 (10, 7–45)
Tumor size group, *n* (%)			
Intracanalicular	40 (6)	40 (7)	0 (0)
Small (0–10 mm)	93 (16)	92 (16)	1 (3)
Medium (11–20 mm)	195 (33)	177 (31)	18 (55)
Moderately large (21–30 mm)	179 (30)	174 (31)	5 (15)
Large (31–40 mm)	72 (12)	65 (12)	7 (21)
Giant (> 40 mm)	17 (3)	15 (3)	2 (6)
Cystic degeneration, *n* (%)			
Cystic	248 (42)	236 (42)	12 (36)
Solid	332 (55)	312 (55)	20 (61)
No data	16 (3)	15 (3)	1 (3)
Preoperative tumor size			
Progressive	187 (31)	173 (31)	14 (42)
Stable	409 (69)	390 (69)	19 (58)
Extent of resection^a^, *n* (%)			
Total	190 (32)	188 (34)	2 (6)
Near total	345 (58)	323 (57)	22 (67)
Subtotal	61 (10)	52 (9)	9 (27)
MRI outcome^b^, *n* (%)			
Residue	183 (31)	155 (28)	28 (85)
No residue	360 (60)	356 (63)	4 (12)
Uncertain	53 (9)	52 (9)	1 (3)
Mean follow-up time, months (median, range)	50 (36, 3–209)	51 (36, 3–209)	47 (39, 19–131)

*SD* standard deviation

^a^As estimated by the surgeon at the end of surgery

^b^As assessed on the first postoperative MRI scan

### Correlation between the extent of resection and postoperative MR imaging

Postoperative estimations of the extent of tumor resection and the occurrence of residual disease as assessed on the first postoperative MRI are listed in Table 1, and linked to recurrence in Table 2. The first postoperative MRI was performed at 11 months on average.

**Table 2:** First postoperative MRI outcome based on the extent of resection

Extent of resection^a^	MRI outcome^b^	All patients, *n* = 596	Recurrence free, *n* = 563	Recurrence, *n* = 33	Months to recurrence, mean (range)
Total	Residue	13 (2)	12 (2)	1 (3)	39
	No residue	154 (26)	153 (27)	1 (3)	131
	Uncertain	23 (4)	23 (4)	0 (0)	–
Near total	Residue	128 (21)	109 (19)	19 (58)	43 (19–88)
	No residue	191 (32)	188 (33)	3 (9)	80 (46–125)
	Uncertain	26 (4)	26 (5)	0 (0)	–
Subtotal	Residue	42 (7)	34 (6)	8 (24)	32 (20–51)
	No residue	15 (3)	15 (3)	0 (0)	–
	Uncertain	4 (1)	3 (1)	1 (3)	56

^a^As estimated by the surgeon at the end of surgery

^b^As assessed on the first postoperative MRI scan

One hundred and ninety patients underwent a total tumor resection. In 154 of these patients (81%), the first postoperative MRI showed no tumor residue; however in 36 patients (19%), the first postoperative MRI showed uncertain enhancement or suspected residual disease. Two out of the 190 patients (1%) developed a recurrence after a perceived total tumor removal.

In 406 patients (68%), the extent of resection was perceived to be near total or subtotal. The first postoperative MRI showed uncertain enhancement or suspected residual disease in 200 of these patients (49%). However, no tumor residue was detected on the first postoperative MRI in 206 patients (51%). Thirty-one patients (8%) developed a recurrence after a less than total tumor resection.

As these numbers indicate, the inter-observation agreement between the estimated extent of resection at the end of surgery and the first postoperative MRI was low (*κ* = 0.134, *p* < 0.001).

### Time to recurrence

The mean follow-up time until the diagnosis of a recurrence was 47 months (median 39, range 19–131 months). The mean time interval to the diagnosis of a recurrence was 85, 49 and 33 months after total, near-total and subtotal resections respectively. In patients with residual tumor on the first postoperative MRI, the mean follow-up time to recurrence was 39 months (*n* = 28, range 19–88 months). Four patients developed a recurrence while their first postoperative MRI did not show residual tumor. The earliest diagnosis of a recurrence in these four patients was established at 46 months of follow-up (Table 2).

### Recurrence prediction model performance

**Fig. 1:**
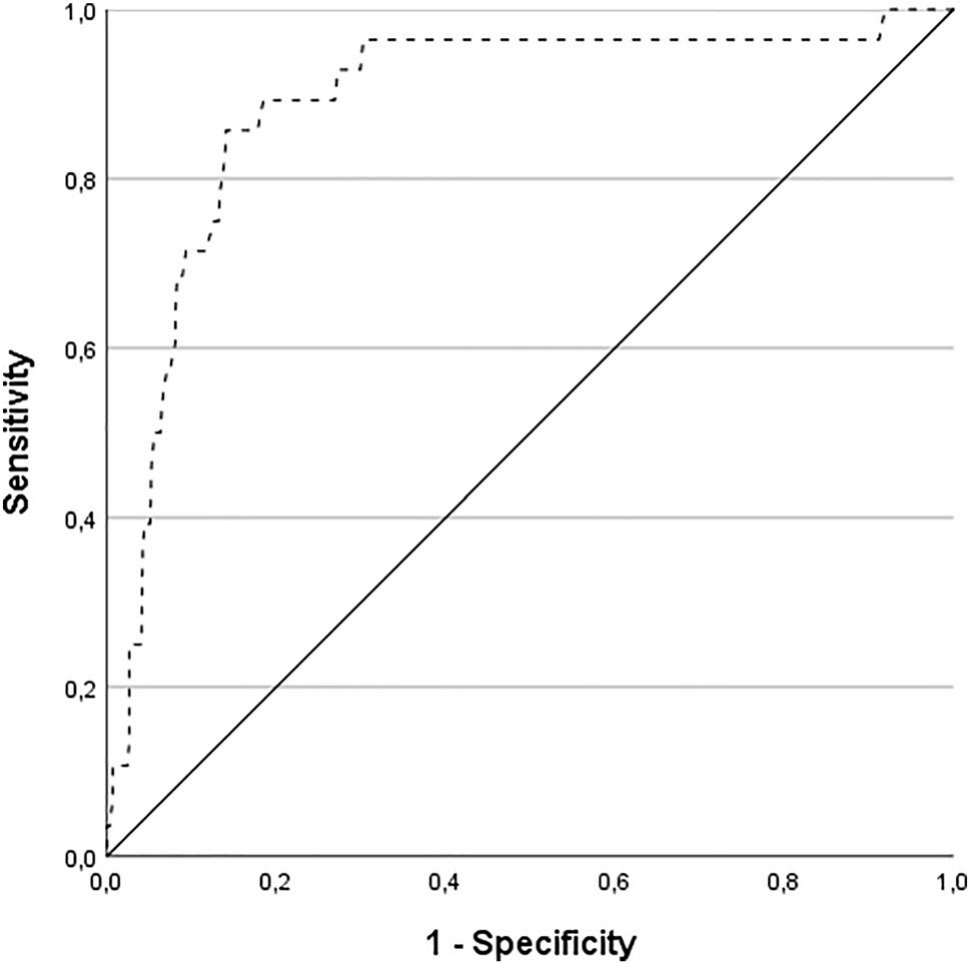
Receiver operating curve of the prediction model for tumor recurrence at 5 years after translabyrinthine surgery for vestibular schwannomas, using the ‘age at time of surgery’, ‘preoperative tumor progression’, and ‘first postoperative MRI outcome’ as predictors. The area under the curve is 89%

First, a Cox regression analysis was performed using all of the potential predictors. A significant correlation on the incidence of a recurrence was found for the first postoperative MRI outcome (*p* = 0.001), the age at time of surgery (*p* = 0.002) and preoperative tumor growth (*p* = 0.042), while no significant association was found for the extent of tumor resection (*p* = 0.279), cystic degeneration (*p* = 0.538), preoperative tumor size (*p* = 0.727) or gender (*p* = 0.358). To prevent overfitting, a model was computed with only three potential predictors by leaving out the non-significant predictors. Saturation of the radiological follow-up data was satisfactory after 5 years postoperatively (199/596 patients) and less adequate after more than 10 years postoperatively (58/596 patients), reflected by a mean follow-up time of 50 months in all patients. Therefore, the ROC of the prediction model was computed on a 5 year postoperative time horizon. The final prediction model consisted of the variables ‘age at time of surgery’ (*p* = 0.003, HR = 0.959 per year of age), ‘preoperative tumor growth’ (*p* = 0.008, HR = 2.715) and ‘first postoperative MRI outcome’ (uncertain enhancement *p* = 0.644, HR = 1.679; no residual tumor *p* < 0.001, HR = 12.063; residual tumor as reference group). These predictors were selected based on their combined performance with AUC comparisons. The ROC of the final prediction model showed an AUC of 89% (standard error [SE] 4, 95% CI 82–95), indicating that the model discriminates well between patients who will develop a recurrence and those who will not (Fig. 1). This model performed similar to the model with all variables (AUC 89%, SE 3, 95% CI 83–95, *p* = 0.558), while using only three predictors. It outperformed a model consisting of the variable ‘first postoperative MRI outcome’ only (AUC 83%, SE 2, 95% CI 79–88, *p* = 0.004), a model consisting of the variables ‘age at time of surgery’, ‘preoperative tumor growth’ and ‘estimated extent of resection’ (AUC 79%, SE 4, 95% CI 72–86, *p* = 0.011), or models based on other combinations of predictors (data not shown). Quantification of the overall performance of the final prediction model, after internal validation, resulted in a cross-validated C-index of 0.686 (95% CI 0.614–0.796), indicating that the model adequately assigns a higher risk score to a patient with an earlier event probability.

### Recurrence prediction model outcome

**Fig. 2:**
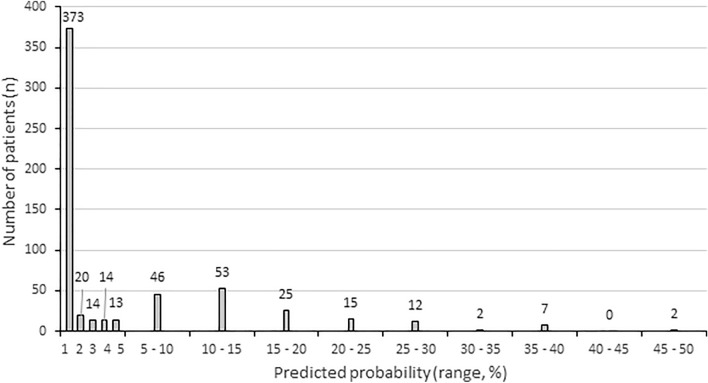
Bar chart showing the number of patients (*y*-axis) per predicted probability group (*x*-axis)

**Fig. 3:**
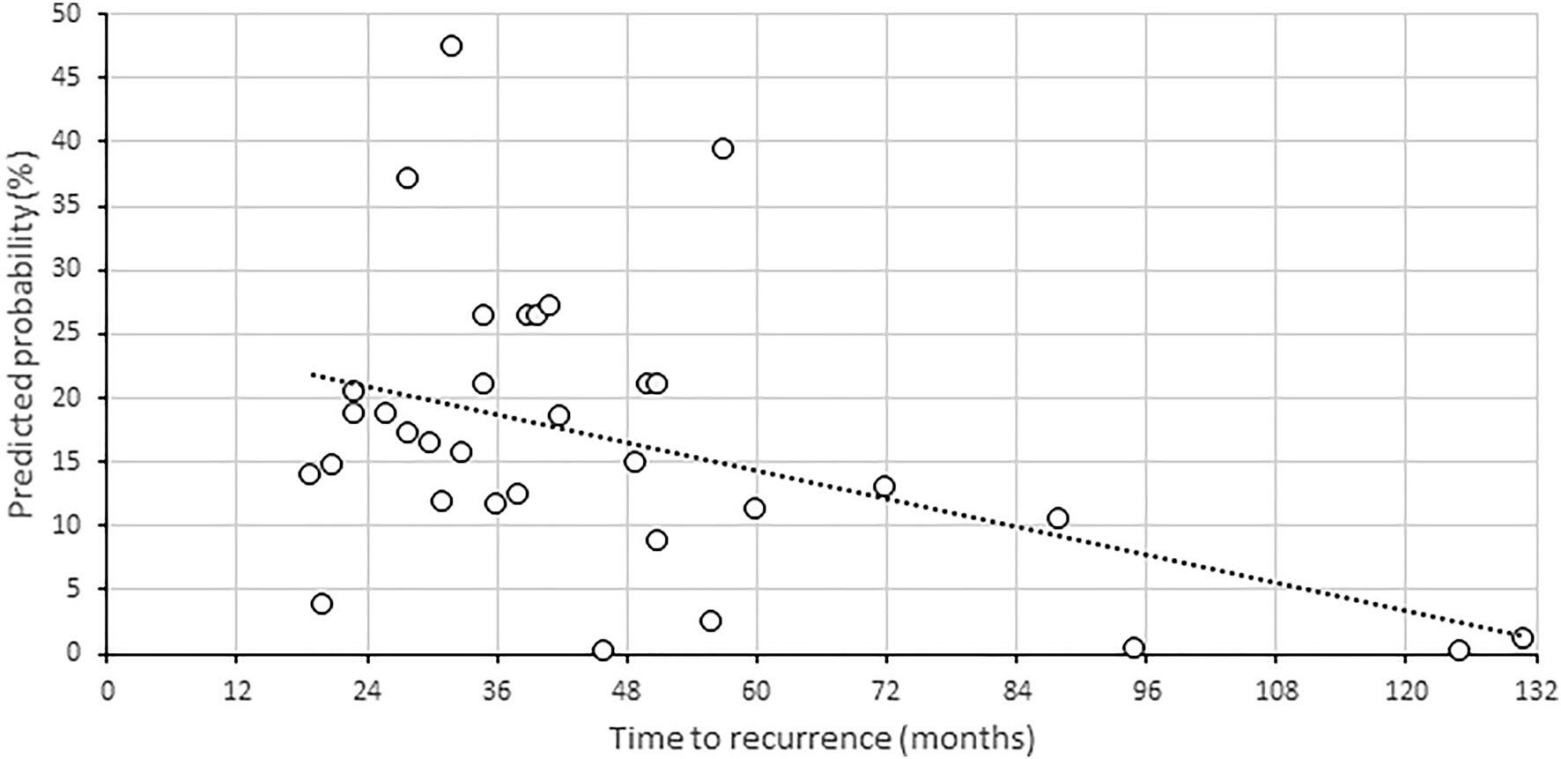
Scatter plot of vestibular schwannomas with recurrence after translabyrinthine surgery (*n* = 33). The plot shows the predicted probability (*y*-axis) of tumor recurrence and time to the diagnosis of recurrence (*x*-axis). The dotted line represents the fit line (trend), indicating that the predicted probability is lower for tumors with late recurrences. In addition, the scatter plot shows that very few (*n* = 3) recurrences occurred in patients with a predicted probability of < 1%, the first 46 months after surgery

Overall, the predicted probability of tumor recurrence at 5 years was < 1% in 373 patients (63%), < 5% in 434 patients (73%), 5–10% in 46 patients (8%), 10–15% in 53 patients (9%) and > 15% in 63 (11%) (Fig. 2). The mean predicted probability in patients without a recurrence was 4.0 (range 0.1–45.1), and 16.6 (range 0.1–47.4) in patients with a recurrence. Within the group of patients suffering from a recurrence, the time to recurrence seems to be longer in patients with a low predicted probability of recurrence (Fig. 3).

## Discussion

Prediction of regrowth of tumor or residual tumor growth following translabyrinthine surgery for vestibular schwannomas is relevant for patient management and planning of additional therapy, should this be required. We developed a model to predict recurrence with a good overall discriminative performance, based on only three variables, i.e., ‘age at time of surgery’, ‘preoperative tumor growth’ and ‘first postoperative MRI outcome’. All three are widely used in clinical practice, objective, and independent of the surgeon’s experience or clinical judgment. As such, this prediction model seems readily applicable in other surgical centers and settings.

### Postoperative follow-up

Saving facial nerve function during surgery is of paramount importance and less aggressive surgical resection of vestibular schwannomas to achieve this has become common practice [20]. The inherent risk of less aggressive surgery, i.e., incomplete tumor resections, is that recurrence rates can potentially increase, making long-term surveillance after vestibular schwannoma surgery all the more important. To date, no universally accepted protocol for postoperative surveillance exists, and surveillance practices vary greatly [9].

As the prediction model presented in the current study calculates risk estimates for individual patients, it can assist in tailoring the postoperative radiological surveillance to the estimated recurrence risk and individualize follow-up strategies. The overall risk of recurrence was 5.5%. Using the prediction model, a significant reduction in the estimated risk of recurrence (to < 1%) was calculated for 63% of all patients. A higher than average risk of recurrence (> 5%) was calculated for 27% of patients. In these ‘intermediate- to high-’ risk patients, we feel that follow-up should be stringent and include regular MRI scans. At our center, these patients undergo follow-up MRI once a year, with increasing time intervals if the tumor remnant remains indolent. We feel that especially in low-risk patients (estimated risk of recurrence < 1%), the interval between the first postoperative MRI and the second follow-up MRI can be prolonged. Three patients (3/373, 1%) in this low-risk group still developed a recurrence, however, only after 46 months (Fig. 3). It seems that in these low risk patients, the time interval between the first postoperative MRI and second follow-up MRI can therefore safely be extended to 3 years, as the risk of recurrence within this time frame is clinically negligible. Minimizing the number of follow-up MRI scans reduces the burden to patients, and as the majority of patients (63%) have a low calculated risk of recurrence, it would also result in a substantial cost reduction.

Two of the three factors in our prediction model, namely ‘age at time of surgery’ and ‘preoperative tumor growth’, are straightforward and not subject to bias. The identification of residual tumor after translabyrinthine surgery on postoperative MR imaging can, however, be challenging. Contrast enhancement on MRI is frequently seen in the first 6 months at the surgical site due to disruption of the blood–brain barrier, neovascularity and/or an inflammatory reaction to the fat graft or bone wax. It may mimic residual tumor and last for years [21–23]. In this series, postoperative enhancement on MRI of uncertain origin occurred in 9% of the patients. Most often, the distinction between postoperative, reactive enhancement and residual tumor can be made using sequential follow-up MRI scans. As recurrences may develop even after perceived total tumor resections, and the time to recurrence may be longer, sequential postoperative follow-up with MR imaging is indicated in all patients undergoing vestibular schwannoma surgery, even in patients with a calculated low risk of recurrence. Therefore, a second postoperative MRI in low risk patients should not be omitted, but the interval between MRI scans may be tailored to the individual patients’ risk profile.

In this study, a discordance between the perceived extent of resection and the outcome of the first postoperative MRI was found, as has been observed previously [6, 8]. The number of patients without evident tumor residue on the first postoperative MRI well exceeds the number of patients with total tumor removal as judged by the surgeon at the end of surgery. A possible explanation for this observation is that the residual tumor mass at the end of surgery is simply too small to be detected on MR imaging. Alternatively, regression of the tumor residue may have occurred postoperatively. Conversely, a larger than expected residual tumor on first postoperative MRI may indicate that precise judgment of the extent of tumor resection by the surgeon can be difficult or it could reflect (re)growth in the interval between surgery and the first postoperative MRI, resulting in an underestimation of the risk of recurrence based on the perceived extent of resection at the end of surgery. Although the surgeon’s assessment of the extent of resection has been reported to be correlated with the risk of recurrence [3], the predictor ‘outcome of the first postoperative MRI’ performs significantly better in the current model (*p* = 0.011), and the performance of the model does not improve with the addition of the surgeon's assessment of the extent of resection. As a postoperative MRI is essential in assessing postoperative status of the surgical site and will be performed within a year regardless of the calculated risk of recurrence, we chose to only incorporate the outcome of this MRI in the model. In addition, this parameter is more objective and better generalizable to other centers and patient cohorts than the extent of tumor resection as judged by the surgeons.

### Limitations

The prediction model for tumor recurrence after vestibular schwannoma surgery presented in this study is based on the experience of one tertiary referral center. The indications and timing of salvage treatment may differ between centers. As the need for salvage treatment defines a recurrence in our center, and a recurrence is the main prediction end point of the current model, the models’ performance may not be universally applicable. Even so, predicting which tumor may need salvage treatment in the future and which may not seems the most clinically relevant end point. Although the models were internally validated, external validation should be performed to evaluate the applicability of the outcome in other centers. Because of the limited number of predictors used in the current model, the generic nature of the predictors and their independence from the surgeon’s experience or clinical judgment, external validity seems likely.

Statistically, the observed data as used in the analysis is interval censored and not right-censored, i.e., only the interval of recurrence (time past last follow-up to current follow-up) is known and not the exact time point of recurrence. Due to slow tumor growth, we believe that the impact of this discrepancy is low. The relatively low number of events prevented us from investigating other deviations from the Cox assumptions, such as non-proportionality or time dependence of effects. Clinically, there are no indications that large effects of such nature would be present.

In addition, in this study a semiquantitative categorization for defining residual tumor on postoperative MR imaging has been used, and not volumetric measurements. However, as volumetrics are notoriously difficult to achieve especially in residual tumors after surgery, a semiquantitative way is most commonly used and thus conforms best to clinical practice.

Last, a cutoff value of 1% for patients at low risk of recurrence seems intuitive and works well in this series, but is inevitably somewhat arbitrary and different cutoff values may better suit the surgical experience and outcomes of other centers.

## Conclusion

This study presents a prediction model for determining the postoperative risk of recurrence after translabyrinthine surgery for individual patients, based on only three widely used, generic clinical parameters: ‘age at time of surgery’, ‘preoperative tumor progression’ and ‘first postoperative MRI outcome’. Especially in patients with a low risk of recurrence (defined as < 1% in this study), one may consider prolonging the interval between the first and consecutive postoperative MRI scans, thereby reducing the number of MRI scans in the first year of follow-up after surgery.

## Data Availability

The data that support the findings of this study are available upon reasonable request from the corresponding author. The data are not publicly available due to their containing information that could compromise the privacy of research participants.

## Abbreviations

AUC: Area under the curve
CI: Confidence interval
HR: Hazard ratio
MRI: Magnetic resonance imaging
ROC: Receiver operating curve
SE: Standard error
